# The Epidemiology and Demographics of Slipped Capital Femoral Epiphysis

**DOI:** 10.5402/2011/486512

**Published:** 2011-09-21

**Authors:** Randall T. Loder, Elaine N. Skopelja

**Affiliations:** ^1^Riley Children's Hospital, Room 4250, 705 Riley Hospital Drive, Indianapolis, IN 46202, USA; ^2^Department of Orthopaedic Surgery, Indiana University, Indianapolis, IN 46202, USA; ^3^Ruth Lilly Medical Library, Indiana University School of Medicine, Indianapolis, IN 46202, USA

## Abstract

The etiology of slipped capital femoral epiphysis (SCFE) is unknown with many insights coming from epidemiologic/demographic information. A systematic medical literature review regarding SCFE was performed. The incidence is 0.33/100,000 to 24.58/100,000 children 8 to 15 years of age. The relative racial frequency, relative to Caucasians at 1.0, is 5.6 for Polynesians, 3.9 for Blacks, and 2.5 for Hispanics. The average age is 12.0 years for boys and 11.2 years for girls. The physiologic age when SCFE occurs is less variable than the chronologic age. The average symptom duration is 4 to 5 months. Most children are obese: >50% are >95th percentile weight for age with average BMI is 25–30 kg/m^2^. The onset of SCFE is in the summer when north of 40°N. Bilaterality ranges from 18 to 50%. In children with bilateral involvement, 50–60% present with simultaneous SCFEs and those who present with a unilateral SCFE and subsequently develop a contralateral SCFE do so within 18 months. The age at presentation is younger for those who present with a unilateral SCFE and later develop a contralateral SCFE. The age-weight, age-height, and height test are useful to differentiate between an idiopathic and atypical SCFE.

## 1. Introduction

Slipped capital femoral epiphysis (SCFE) is an adolescent hip disorder with displacement of the capital femoral epiphysis from the metaphysis through the physis. Most SCFEs are varus (medial and posterior epiphyseal displacement relative to the metaphysic) but can occasionally be valgus (lateral and superior epiphyseal displacement) [1–4]. The vast majority are “idiopathic”; atypical SCFEs are those that occur due to an endocrine disorder [5–7], renal failure osteodystrophy [8], or radiation therapy [7, 9, 10]. The literature regarding the epidemiology and demographics of SCFE first requires a general knowledge of the disorder. 

SCFE is classified both clinically and by severity. The traditional clinical classification was acute, chronic, and acute on chronic [12–16], based on the patient's history, physical examination, and roentgenograms. An acute SCFE is defined as those with symptoms for <3 weeks with an abrupt displacement through the proximal physis [12]. Chronic SCFEs present ≥3 weeks of symptoms and account for 85% of all SCFEs [17]. Acute-on-chronic SCFEs are those with chronic symptoms initially and the subsequent development of acute symptoms. Newer classifications are more clinically useful, depend upon physeal stability, and predict the prognosis regarding subsequent avascular necrosis. There are two such classifications: one clinical and one radiographic. The clinical classification depends upon the ability of the child to ambulate [18]. A stable SCFE is defined as one where the child is able to ambulate, with or without crutches. An unstable SCFE is defined as one where the child cannot ambulate, with or without crutches. The radiographic classification depends upon the presence or absence of a hip effusion on ultrasonography [19, 20]. The absence of metaphyseal remodeling and the presence of an effusion define an unstable SCFE; metaphyseal remodeling and the absence of an effusion define a stable SCFE. Unstable SCFEs have a much higher incidence of avascular necrosis (up to 50% in some series) compared to stable SCFEs (nearly 0%) [18]. 

**Figure 1:**
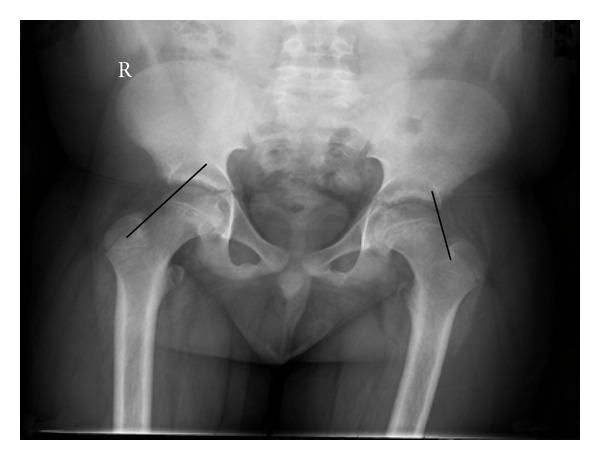
A normal and abnormal epiphyseal line as described by Klein et al. [11] in an 11 year 6 month old boy with a left SCFE. In this anterior-posterior pelvis radiograph proximal prolongation of the superior neck line transects the epiphysis in the normal hip (right) but either lies flush with or does not transect the epiphysis in SCFE (left hip).

Radiographs demonstrate an inferior and posterior slip of the proximal femoral epiphysis relative to the metaphysis. The gradual slip demonstrates radiographic signs of remodeling on the superior and anterior femoral metaphysis, and periosteal new-bone formation at the epiphyseal-metaphyseal junction posteriorly and inferiorly. In the early slip, the changes can be subtle with only posterior displacement [21]. As such it is usually seen early only on the lateral view, and both anteroposterior and lateral radiographs must be obtained. Other radiographic signs of an early SCFE are the metaphyseal blanch sign of Steel [22] and Klein's line [11]. The metaphyseal blanch sign of Steel is a radiographic double density seen on the anteroposterior view at the level of the metaphysis; this double density reflects the posterior cortical lip of the epiphysis as it is beginning to slip posteriorly and is radiographically superimposed upon the metaphyseal density. Klein's line is drawn along the anterior or superior aspect of the femoral neck; the epiphysis should normally intersect this line. In an early SCFE the epiphysis will be flush with or even below this line (Figure 1).

**Figure 2:**
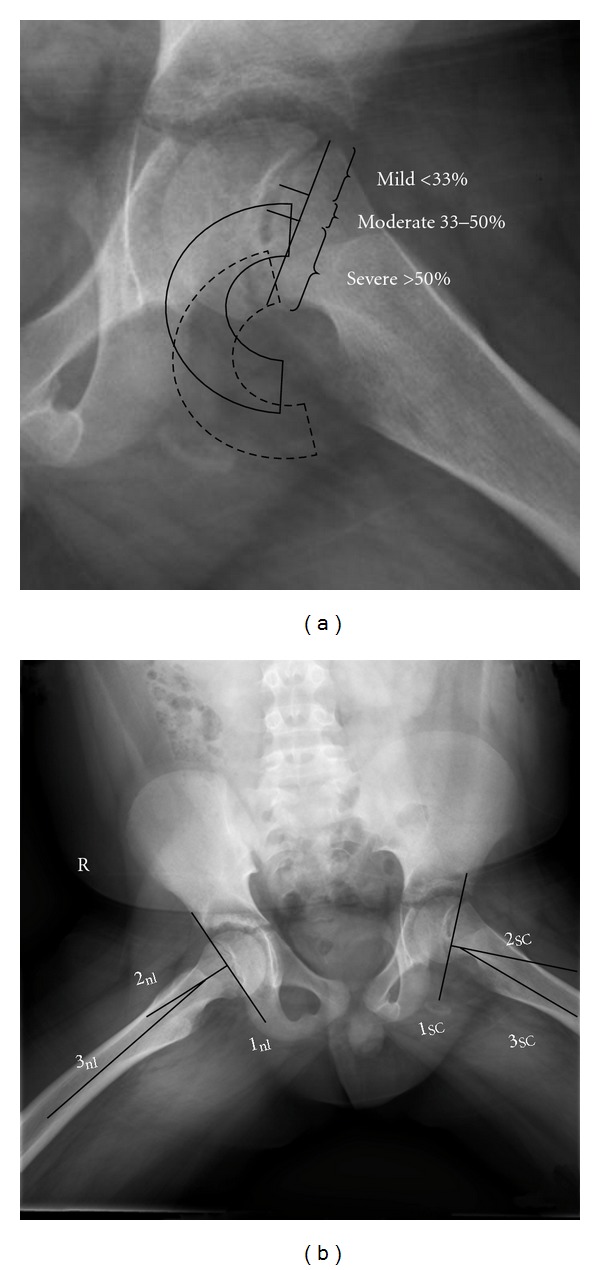
The two different methods of assessing SCFE severity. (a) The amount of displacement of the epiphysis relative to the metaphysis. A mild SCFE is defined as epiphyseal-metaphyseal displacement <1/3 the width of the metaphysis; a moderate SCFE 1/3–1/2; a severe SCFE as >1/2 [23]. In this case the SCFE is mild. The position of the epiphysis in a moderate SCFE is represented by the solid semicircle and in a severe SCFE by the hatched semicircle. (b) The lateral epiphyseal shaft angle measurement as described by Southwick [25] on the frog lateral radiograph. The frog lateral radiograph of the case in Figure 1 is shown. Both the normal hip (nl) and SCFE hip (SC) are measured. Line 1 is the line between the anterior and posterior physis, line 2 is a perpendicular to line 1, and the intersection of line 2 with an axial line along the shaft of the femur (line 3) is the epiphyseal shaft angle. The slip angle is calculated by subtracting the epiphyseal shaft angle of the normal hip from the slip side. Those less than 30 degrees are considered mild, between 30 and 50 degrees moderate, and greater than 50 degrees severe. The severity of the SCFE is the lateral epiphyseal shaft angle of the normal hip subtracted from the SCFE hip. In this example the slip angle is 25°–10° or 15°.

SCFE severity is described by two different methods. The first is the amount of displacement of the epiphysis on the metaphysis (Figure 2). A mild SCFE is defined as epiphyseal-metaphyseal displacement <1/3 the width of the metaphysis; a moderate SCFE 1/3–1/2; a severe SCFE >1/2 (Figure 2(a)) [23]. This method is less accurate than angular measurement since distinct landmarks are difficult to determine due to metaphyseal remodeling in the gradual stable SCFE [24]. Angular measurement uses the epiphyseal-shaft angle on the frog-lateral radiograph [25] (Figure 2(b)) and is categorized into 3 groups: mild <30°, moderate 30–50°, and severe >50° [16]. This classification is important for long-term prognosis as mild and moderate slips have a much better long-term prognosis than severe slips which demonstrate a more rapid development of degenerative hip disease [26, 27]. CT scan measurements are no more accurate than those using conventional radiographs [28]. 

## 2. Materials and Methods

A systematic review of SCFE was performed. SCFE has been known by many different names since its first description in the late 19th and early 20th centuries. Prior to the last 40 years or so, the accepted name for this condition had been very inconsistent. Even now there is no actual MESH (Medical Subject Heading) term for slipped capital femoral epiphysis (SCFE). Users must combine “epiphyses, slipped” and “femur head”. The modern, most used, frequently used term “slipped capital femoral epiphysis” or SCFE, must be searched as a keyword phrase. To ensure capture of all the published literature, older terms were also searched as keywords or keyword phrases. Therefore the terms used to search for SCFE were slipped capital femoral epiphysis, slipped epiphysis, epiphysiolysis, and epiphysiolyses. The last strategy was to combine these two sets of terms: (slipped OR slipping OR sliding OR displaced OR displacement OR detached OR detachment OR separated OR separation) combined with (femur OR head OR capitis OR epiphysis OR epiphyseal OR epiphysiolysis OR epiphysiolyses).

The databases searched were PubMed (1947–2010) (http://www.ncbi.nlm.nih.gov/pubmed/), Ovid Medline (1947–2010), EMBASE (1947–2010), WorldCat (1880–2010) (books and theses) (http://firstsearch.oclc.org/), Google Scholar (1880–2010) (http://scholar.google.com/), Web of Knowledge (1987–2010), and IndexCat (1880–1961) (Index Catalogue of the Library of the Surgeon-General's Office) (http://www.indexcat.nlm.nih.gov/). Exclusion criteria were those manuscripts discussing surgery, therapy, rehabilitation, and any foreign language articles without an English abstract. Individual orthopaedic journals were also searched for articles published prior to 1966 that predate electronic Medline indexing, including *Journal of Bone *and* Joint Surgery (American *and* British), Clinical Orthopaedics *and* Related Research*, and *Acta Orthopaedica Scandinavica*. Age groups were limited to those <18 years old; duplicate citations were removed. 

This search resulted in 1407 unique citations. These 1407 manuscripts were reviewed to find those that discussed any of the topics regarding SCFE and epidemiology, demographics, incidence, prevalence, race, gender, family history, inheritance, genetics, age, bone age, weight (either birth weight or normal weight), height, growth, maturation, any other anthropometric characteristics, seasonal variation, hormone, endocrine, congenital anomalies, collagen, immunoglobulin, and opposite hip. Of these 1407 manuscripts, 114 provided ample information and are the contents of this paper.

## 3. Results

### 3.1. Incidence

Conventional quotation for SCFE incidence in the literature is the number of cases per 100,000 (usually for the age range 8 to 15 years old, although some use <25 years of age) and is used throughout this paper. The incidence (Table 1) ranges from 0.2 per 100,000 in eastern Japan [36] to 17.15 in the Northeastern United States [31]. Recent studies indicate that the overall incidence in the United States is 10.8/100,000 [31], similar to 10.08 in boys in the Kelsey et al.'s study [37]. In Japan it has increased to 2.22/100,000 for boys and 0.76/100,000 for girls [38]. In Olmsted County, Minnesota, the incidence was 8.8; 8.3 for unilateral cases and 0.5 for bilateral cases [39]. The incidence is increasing in some areas compared to previous reports; in New Mexico [32] it has nearly tripled (6.05 versus 2.13) compared to an earlier study [37]. In Scotland, the incidence has increased from 3.78 in 1981 to 9.66 in 2000, correlated with rising obesity [40]. The highest presently quoted incidence is in US Africans at 24.58 [31].

There is little data regarding differences between urban and rural areas. The incidence of SCFE in southern Sweden increased in rural areas for boys (7.5 versus 4.8) but decreased for girls (2.2 versus 3.8) [34]. No differences were noted in Connecticut in white children between urban and nonurban settings (3.19 for both) [41] although there was an increased incidence in Africans living in urban areas compared to nonurban areas (7.95 versus 1.35). No differences were noted by gender.

In the USA there are differences by geographic region. In a recent study [31] the highest incidence was in the Northeast at 17.15 and the lowest in the Midwest at 7.69. This is similar to an earlier study [37] where the incidence in the Northeast was 10.08 and 2.13 in New Mexico.

### 3.2. Race/Ethnicity

**Figure 3:**
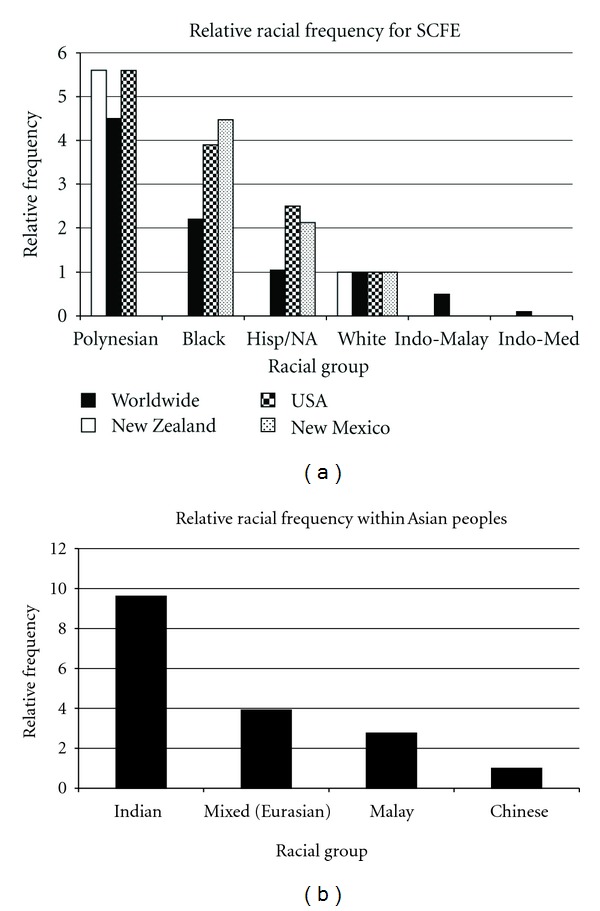
(a) Relative racial frequencies of SCFE normalized to Caucasian children. The New Zealand data is from [29], worldwide data from Loder [30], USA data from Lehmann et al. [31], and New Mexico data from Benson et al. [32]. (b) Relative racial frequencies of SCFE within the Indo-Malay and Indo-Mediterranean groups, normalized to Chinese children. Data from [33] was used to calculate the relative racial frequencies using previously described methods [30].

There is significant racial variability with SCFE (Table 2). Race is classified using the definitions of Eveleth and Tanner: Caucasians, Africans in Africa and of African Ancestry, Asiatics (Amerindians, Hispanics, Indonesian-Malays), Indo-Mediterraneans (inhabitants of the Near East, North Africa, and Indian subcontinent), and Australian Aborigines and Pacific Island peoples [53]. The relative racial frequency (RRF) of SCFE, with Whites at 1.0, is 4.5 for Pacific Islanders, 2.2 for Africans, 1.05 for Amerindian (Native Americans and Hispanics), 0.5 for Indonesian-Malay (Chinese, Japanese, Thai, Vietnamese, etc.), and 0.1 for Indo-Mediterranean peoples (Near East, North African, or Indian subcontinent ancestry) [30]. More recent data indicates that these numbers, relative to Caucasians, is 5.6 for Polynesians, 3.9 for Blacks, and 2.5 for Hispanics (Figure 3(a)) [29, 31]. In New Zealand the RRF was 5.6 for the Maori peoples and 4.2 for other Pacific Islanders [29]. In Singapore the RRFs, relative to Chinese, are 9.6 for Indo-Mediterraneans (Indian), 2.8 for Malay, and 3.0 for mixed (Eurasians) (Figure 3(b)) [33]. Thus Indo-Mediterraneans (Indian) had a 9-fold increased incidence compared to Indo-Malay (Chinese). This is in contrast to another study [30] where Indo-Mediterraneans had a 5 times lower frequency of SCFE compared to Indo-Malays (all groups—Japanese, Thai). These racial differences most likely reflect the average body weights for each racial group and further support the significant role that obesity and mechanical stress plays in the etiology of SCFE [30]. A less likely explanation is racial variability in acetabular depth and femoral head coverage; the acetabulae in African children were deeper than White children in one study [54], but not in another study [55]. 

### 3.3. Gender and Age

Most series demonstrate a male predominance. Early in this century 90% occurred in males [57] but has now decreased to ~60%. In a review of 4343 children, 64.3% were boys and 35.7% girls (Table 3). There are differences in gender by race, with Indo-Mediterraneans having the highest proportion of boys (90%) and Polynesians an equal male/female ratio [30].

**Figure 4:**
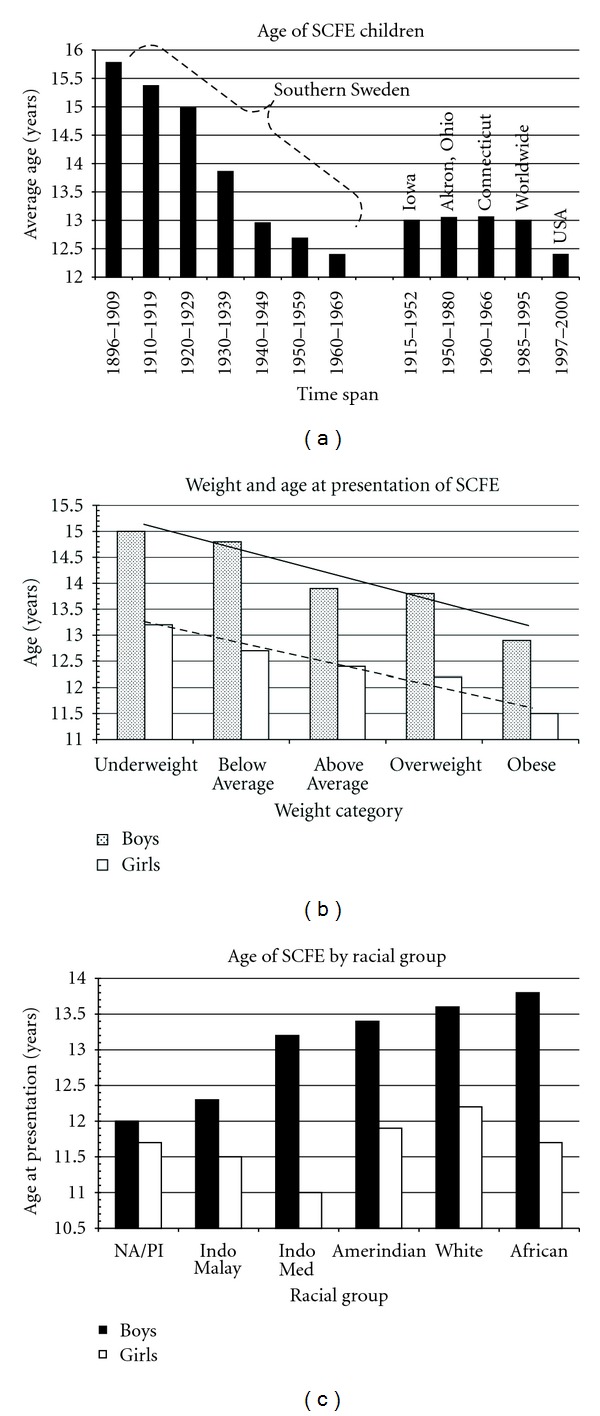
(a) The changing age at diagnosis of children with SCFE over the last century. The southern Sweden data is from [34]; the Iowa data is from [26]; the Akron, Ohio data is from [35]; the worldwide data is from Loder [30]; the USA data from Lehmann et al. [31]. (b) The average age at presentation of children with SCFE by weight category for both girls and boys. The average age follows a linear with the weight category (WC) for both the boys (solid black line) (age = 12.5−0.52 WC, *r* ^2^ = 0.94, *P* = 0.006) and girls (hatched black line) (age = 11.2−0.39 WC, *r* ^2^ = 0.96, *P* = 0.003) {WC of 5 = obese class, WC of 4 = overweight class, WC of 3 = above average class, WC of 2 = below average class, and WC of 1 = underweight class}. Data from Loder [30]. (c) The average age by racial group for girls and boys with SCFE. NA/PI: Native Australian/Polynesian, data from Loder [30].

**Figure 5:**
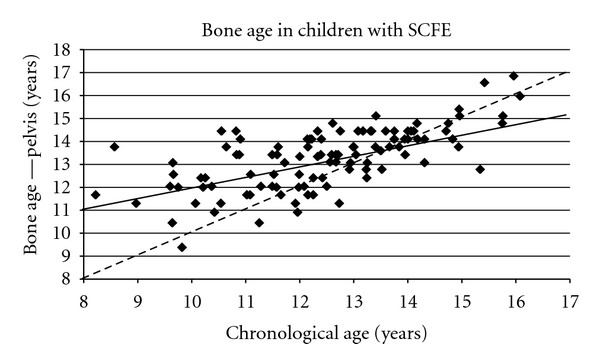
The pelvis bone age (Oxford score converted into years) as a function of chronologic age in 108 children with SCFE. The observed pelvis bone age is represented by the solid line, and is represented by the equation Pelvis Bone Age = 6.95 + 0.51 (Chronologic Age), *r* ^2^ = 0.47, *P* < 10^−6^. The dotted line represents what would be found if the pelvis bone age and chronologic age were the same. The slope of the linear correlation for the actual pelvis bone age, 0.51, is 1/2 that if the bone and chronologic ages were equal, supporting the concept of the narrow bone age window for proximal epiphyseal slipping, data from Loder et al. [56].

SCFE is a disease of prepubescence and early adolescence. Early in this century the average age was much higher with a gradual decrease over time (Figure 4(a)). Fifteen years ago the average age was 13.5 years for boys and 12.0 years for girls [30] and is now 12.0 years for boys and 11.2 years for girls (Table 3). In Scotland, the age has dropped from 13.4 to 12.6 years over a 20-year period [40]. These younger ages reflect the earlier maturation of today's children [31]. Obese children present earlier than nonobese children (Figure 4(b)). One study demonstrated different ages at presentation by racial group (Figure 4(c)) [30]. The physiologic age range during which SCFE occurs is less variable than the chronologic age range (Figure 5), termed the “narrow window” [56, 63].

### 3.4. Symptom Duration

The average symptom duration for stable SCFEs is 4 to 5 months (with minimal difference by gender). In 2482 children, the average symptom duration was 4.3 months; 4.5 months for boys and 3.6 months for girls (Table 4). Two recent studies have noted a decrease in symptom duration to 2-3 months [46, 88]. 

**Figure 6:**
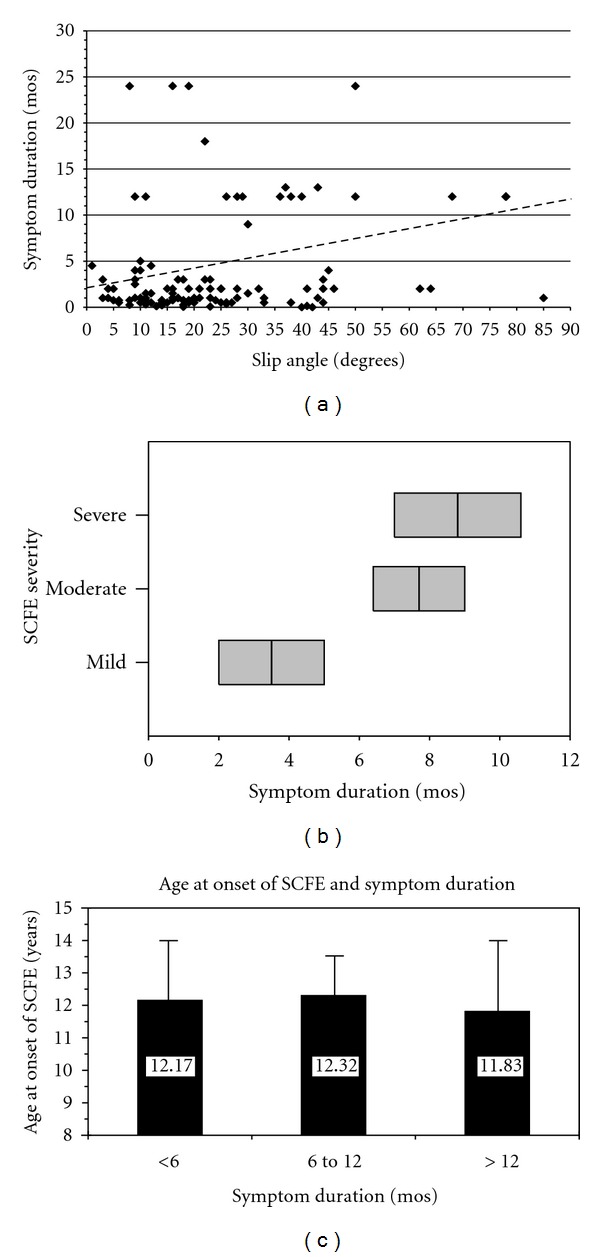
(a) Symptom duration as a function of slip severity (lateral epiphyseal shaft angle) in 254 stable SCFEs. The best fit line is represented by the dotted line: symptom duration = 2.16 + 0.106 (slip angle), (*P* < 0.0001, *r* ^2^ = 0.084), where the slip angle is expressed in degrees and the symptom duration in months. Data from Loder et al. [46]. (b) Average symptom duration in months (middle black line in the gray box) ± one standard deviation (entire gray box) for mild, moderate, and severe stable SCFEs, data from Loder et al. [46]. (c) Categories of symptom duration in months (solid column) + one standard deviation (error bar) and age at onset of the SCFE defined as age at diagnosis minus symptom duration. There was no statistically significant difference in the age at onset for those children with different categories of symptom duration (ANOVA_2, 258_, *F* = 0.56, *P* = 0.57), indicating a small age window at which the SCFE begins. The average age for each symptom duration group is noted, data from Loder et al. [46].

Although there is a general correlation between symptom duration and SCFE severity [88] there is considerable variability (Figure 6(a)) [46]. For any given individual child, slip severity and symptom duration is unique; in a large population, there is a weak positive correlation with slip severity and symptom duration. Mild SCFEs have a shorter duration of symptoms than severe SCFEs (Figure 6(b)) which suggests that all SCFEs start at a similar age; those children with severe SCFEs just had a longer symptom duration and time to presentation than those with milder SCFEs. In a review of 328 stable SCFEs this was documented; a child with a stable SCFE was 2.0 times more likely to have a moderate or severe SCFE if > than 12.5 years at diagnosis than if <12.5 years at diagnosis, and a child with a stable SCFE is 4.1 times more likely to have a moderate or severe SCFE if the duration of symptoms is > than 2 months than if ≤ 2 months [46]. Other positive predictors of increasing time to diagnosis are knee/distal thigh pain, Medicaid coverage, lower family income, and a stable SCFE [88]. 

Slip severity and symptom duration is greater in children presenting with knee pain compared to other symptoms [88–91]. In a study of 45 children [89], 15 presented solely with knee pain having an average symptom duration of 8.3 months; 74% of the SCFEs were severe. The remaining 30 presented with other symptoms with an average symptom duration of 6.5 months; 24% of the SCFEs were severe. Rahme et al. [90] reviewed 87 children with stable SCFEs, 20 had a delayed diagnosis, with 8 (40%) being severe. In Leeds, England, those children with SCFE diagnosed in the emergency department compared to general practitioners had a shorter symptom duration (95 versus 119 days) and fewer severe SCFEs (8 versus 21%). One study, however, did not see a difference in symptom duration in SCFE children where the diagnosis had been initially missed due to the absence of hip pain (average symptom duration 127 days and 146 days in those whose diagnosis was and was not missed) [92].

It would seem reasonable that as symptom duration increases radiographic changes should concomitantly progress (more metaphyseal remodeling). The presence or absence of a positive Klein line and metaphyseal remodeling (superior and anterior smoothing, inferior and posterior buttressing) were directly correlated to symptom duration but with significant variability [93]. Those with more metaphyseal changes had higher BMIs, interpreted as the larger body mass per unit height resulting in more bony reaction/adaptation according to Wolf's law. 

The age at diagnosis of the 1st SCFE correlates with SCFE severity and is also related to the narrow physiologic window [56, 63]. Subtracting the average symptom duration for the mild, moderate, and severe categories from the average age at presentation results in a similar average age at onset (Figure 6(c)), confirming the narrow bone age window.

### 3.5. Body Weight, Obesity, and BMI

The majority of children are obese; >50% of children with SCFE are >95th percentile weight for age [30, 94–96]. Age at diagnosis decreases with increasing obesity [30]; 12.4 years for those over the 95th percentile weight for age and 14.3 years for those under the 10th percentile weight for age. Recently body mass index (BMI) [97, 98] has been used to evaluate body habitus; the average BMI in SCFE children is 25–30 kg/m^2^ or >85th percentile [39, 46, 99–102]. The average BMI of children with bilateral SCFE is higher than with unilateral SCFE (26.8 versus 31.1 kg/m^2^) [99].

Obese children have decreased femoral anteversion, and SCFE children have femoral retroversion even in the noninvolved hip [103, 104]. This, along with biomechanical studies demonstrating an increased physeal stress in retroverted hips and the many demographic studies associating obesity with SCFE, supports the theory that obesity is intimately involved in the development of most idiopathic SCFEs [105].

### 3.6. Seasonal Variation

**Figure 7:**
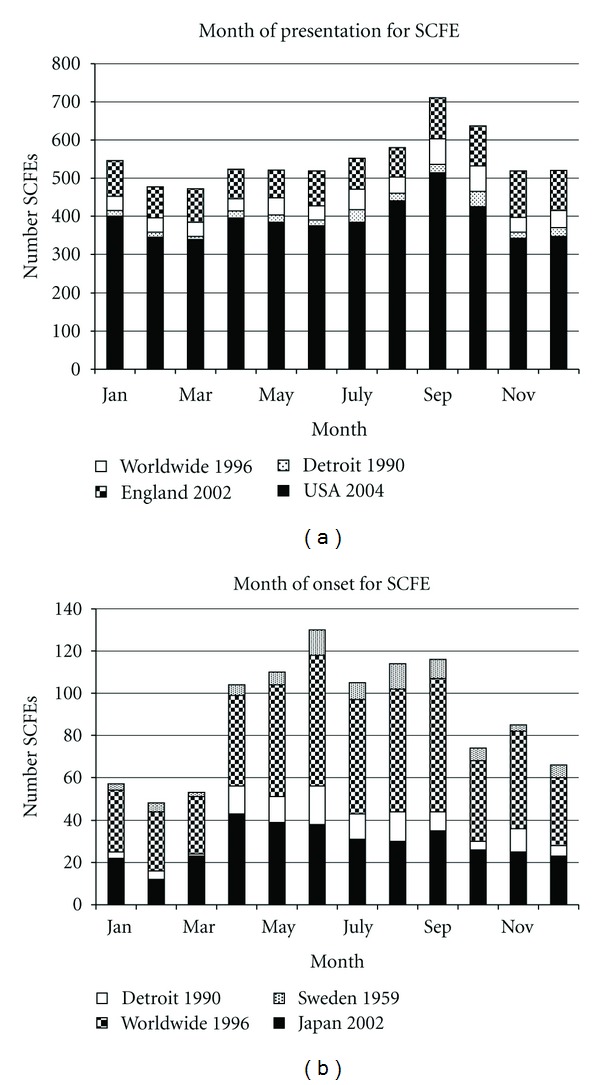
(a) Seasonal variation in the month of presentation of children with SCFE when north of the 40° North latitude. The data for England 2002 are from Maffulli and Douglas [58], worldwide 1996 from Loder [59], Detroit 1990 from Loder et al. [60], and USA 2004 from Brown [61]. Note the peak in September and October. (b) Seasonal variation in the month of onset of children with SCFE when north of the 40° North latitude. The data for Sweden 1959 are from Andrén and Borgström [62], worldwide 1996 from Loder [59], Detroit 1990 from Loder et al. [60], and Japan 2002 from Noguchi and Sakamaki [38]. Note the peak in June, ~3 to 4 months before the peak for presentation in (a).

In latitudes north of 40° SCFE presents more frequently in the late summer and autumn months (Figure 7(a)) [34, 58, 59, 61, 62, 106, 107]. Subtracting symptom duration from the time of presentation demonstrates that the SCFE onset is in the early summer/late spring months (Figure 7(b)).

### 3.7. Bilaterality

The reported proportion of bilaterality depends upon the study, method of radiographic measurement, race, and treatment (Table 5). Most series report 18 to 50% bilaterality [30] with recent follow-up studies into adulthood quoting 63% [34, 109, 110] and 66% [111]. Bilaterality is more frequent in Africans (34%) than Hispanics (17%), Whites (17%), or Asians (18%) [30]. Treatment may affect risk of bilaterality; bilaterality is 36% with *in situ* fixation and 7% spica cast treatment [112]. Close attention is mandatory to the opposite normal hip in those children with a unilateral SCFE treated by *in situ* fixation. 

The age at presentation is younger in children who present with a unilateral SCFE that later develops bilateral SCFEs compared to those who do not develop bilateral SCFEs [17, 50, 59, 113–116]. This age difference is seen in both chronologic age (12 versus 13 years of age) [17, 30, 115, 116] and bone age. In one study the overall difference in chronologic age in those with a unilateral SCFE that became bilateral compared to those that stayed unilateral was 1 year (12 versus 13 years) [17]; in another study 0.9 years for girls (11.0 versus 11.9 years) and 2.2 years (12.1 versus 14.2 years) for boys [115]; and in a 3rd study 11.5 versus 12.7 years, with those children aging <12 years having a unilateral SCFE demonstrated a 3.8 times increased risk of developing a contralateral SCFE [116]. In a study of 50 children with unilateral SCFE [113] using a modified Oxford hip bone age, a contralateral SCFE occurred in 85% of hips having a score of 16, 11% of hips having a score of 21, and never when the score was ≥22. Once the triradiate cartilage has closed, the risk of a contralateral SCFE is only 4% [117], although another study did not find any association between the status of the triradiate cartilage, other skeletal maturity markers, and subsequent bilaterality [118]. The risk of bilaterality is increased when the posterior slope angle of the capital femoral epiphysis is higher [119, 120]. 

**Figure 8:**
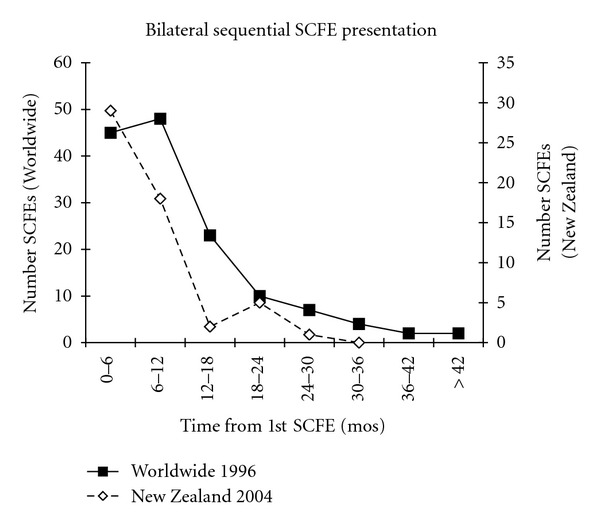
The time between the diagnosis of the 1st and 2nd SCFE of children with sequential bilateral SCFEs. The number of bilateral SCFEs for every 6 month time period from the time of the 1st SCFE is shown. The worldwide data are from Loder [30], and New Zealand data from Stott and Bidwell [29].

In children with bilateral SCFEs, 50–60% present with simultaneous bilateral involvement. In those children with sequential bilateral SCFEs, 80–90% of the second SCFEs occur within the first 18 months after the first SCFE (Figure 8) [17, 29, 30, 34, 50, 67, 102, 121, 122]. In unilateral SCFEs, 60% involve the left hip.

### 3.8. Predictors of Idiopathic and Atypical SCFEs

Tests for a disease are evaluated in terms of sensitivity, specificity, positive predictive value, and negative predictive value [123]. Sensitivity is the proportion of individuals in a tested population who actually have the disease and are identified as having it with the test. Specificity is the proportion of individuals in a tested population who do not have a given disease and are identified as not having it with the test. Sensitivity is increased at the expense of specificity. The probability that a person with a positive result truly has the disease is the positive predictive value; the probability that a person with a negative test truly does not have the disease is the negative predictive value. When evaluating a child with a newly diagnosed SCFE, the underlying etiology immediately comes into consideration. This is for both diagnostic concerns with potential medical issues as well as orthopaedic treatment. The underlying medical issues involve significant anesthetic concerns [108]; treatment involves the question of prophylactic fixation of the opposite hip when the child presents with a unilateral SCFE. Any “test” that can give an accurate negative predictive value for a child with a SCFE not having an atypical SCFE is important. If the test has a high negative predictive value, the clinician can be confident that the SCFE is idiopathic. If the test is positive, further diagnostic investigation should be strongly considered. 

**Figure 9:**
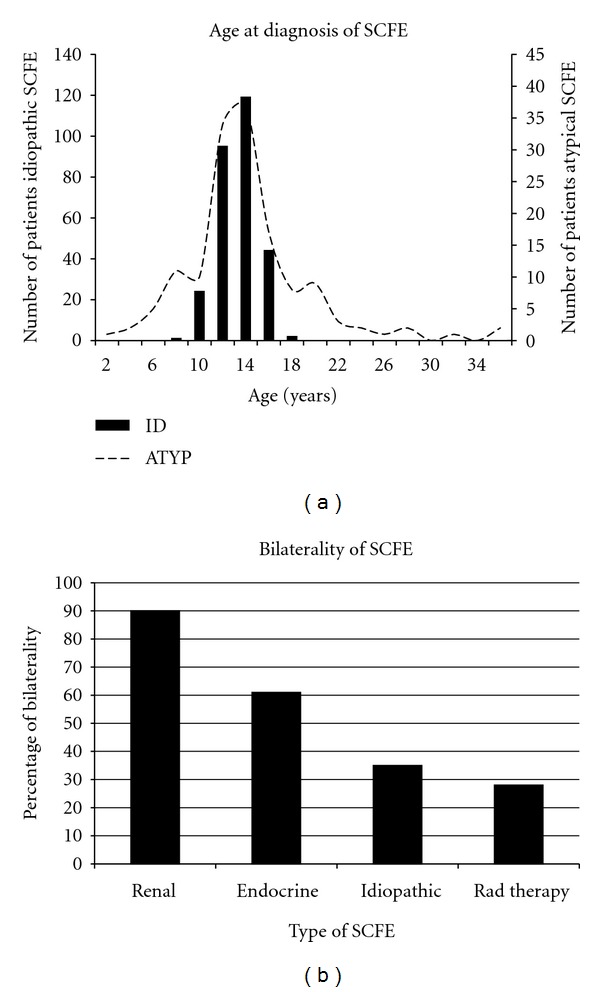
(a) A histogram showing the age at diagnosis of SCFE. Note the narrow age range for children with idiopathic SCFE (92% 10–16 years of age), with a broader range for children with atypical SCFEs (those associated with renal failure, radiation therapy, or endocrinopathy). Data from the study of Loder and Greenfield [108]. (b) Frequency of bilaterality amongst different types of SCFE, data from Loder and Greenfield [108].

Atypical SCFEs are those associated with endocrine/renal disorders or prior radiation therapy. The history of prior radiation therapy is usually discovered in the initial evaluation. However, many times it is the orthopaedic surgeon who picks up on an underlying medical problem as the etiology of the SCFE (e.g. endocrine dysfunction, renal osteodystrophy). In 2001 the age-weight test [108] was described to assist in the differentiation between an idiopathic and atypical SCFE. The age-weight test is defined as negative when age <16 years and weight ≥50th percentile and positive when beyond these boundaries (Table 6). The “disease” is the child having an atypical SCFE, absence of “disease” is the child having an idiopathic SCFE. The demographics of 433 children (285 idiopathic, 148 atypical) with 612 SCFEs were studied to define predictors of atypical SCFEs. There was significant variability in both age and bilaterality between the atypical and idiopathic groups (Figure 9). Multiple logistic regression analysis demonstrated that age and weight were predictors of an atypical SCFE. For two patients of equal weight, those <10, or >16 years of age are 4.2 times more likely to have an atypical SCFE; for two patients of equal age, those <50th percentile weight are 8.4 times more likely. The probability of a child with a negative test having an idiopathic SCFE is 93%, and a child with positive test having an atypical SCFE is 52%. In the same year the age-weight test was described for all atypical SCFEs, Burrow et al. [124] showed that the height at diagnosis was an important predictor of an endocrinopathy. The sensitivity and negative predictive value of detecting an underlying endocrinopathy in a patient with a SCFE who was <10th percentile in height for age were 90.2% and 98%. 

The age-weight test was further confirmed in 2006, along with the definition of the height and age-height tests [125]. The age-height test was defined using the same cells as the age-weight test except that the percentiles applied to height rather than weight. This test has a positive and negative predictive value of 30% and 98%. The height test is defined as positive if the child's height is ≤10th percentile and negative if >10th percentile for age. It is the same test described by Burrow et al. [124] amplified to include all atypical SCFEs, not just those associated with an endocrinopathy. The height test has a positive and negative predictive value of 75% and 97%. 

Of these three tests, all have similar negative predictive values (93 to 98%). The height test has the best positive predictive value (75%). The height test is likely to be the most useful in the differentiation between a typical and atypical SCFE if the height of a child can be obtained. When the height cannot be obtained, the age weight test will result in a similar negative predictive value but with a lower positive predictive value. Thus the weight, and where possible, the height of any child newly diagnosed with a SCFE should be obtained to assist in the differentiation between an atypical and idiopathic SCFE.

### 3.9. Inheritance and Genetics

Little is known regarding the heredity and genetics of SCFE. Familial SCFE was first noted in the English language literature in 1940 [64] with many more subsequent descriptions [65–74, 76–80, 83–87]. Proposed patterns of genetic transmission include X-linked dominant, autosomal dominant with variable penetrance, and autosomal recessive [68, 71, 73, 77]. HLA phenotype studies in children demonstrate no common findings (Table 7) [74, 75, 79–82, 86].

### 3.10. Miscellaneous Associations/Findings

**Other Skeletal Abnormalities**

In most children with SCFE, femoral anteversion is decreased. In a 3-dimensional CT scan study of 30 SCFEs [126] there was a reduction in femoral anteversion from 12.7° to 7.0° as well as a reduction in the femoral neck shaft angle from 141° to 134°. In another CT scan study of 25 SCFEs [103] a reduction of femoral version 1° was noted in the involved hip and 7° in the uninvolved hip in unilateral cases. In a 3rd CT scan study of 25 SCFEs [127] femoral anteversion was 9.8° and 25° in a control group of children between the ages of 8 and 16 years. Reduced femoral anteversion in obese adolescents is well known [104]. Patients with acute SCFEs have less reduction in anteversion compared to those with chronic SCFEs (9.3° versus −0.7°) in the involved hip and uninvolved hip (15.7° versus 11.8°), with an average difference in version in acute SCFEs of 6.7° and in chronic SCFEs of 13.9° [128]. SCFE has no influence on acetabular development [129, 130]. There are also no abnormalities in tibial torsion in children with stable SCFEs [131].

Genu recurvatum has been fleetingly described in SCFE [132, 133]. It is believed that this represents a gradual anterior slip of the proximal tibial epiphysis, with the physes perhaps universally weak or susceptible to large stresses from obesity. Surprisingly, tibia vara (Blount's disease) is very rarely associated with SCFE [134–136] even though both conditions typically occur in obese children. Peroneal spastic flatfoot has been associated in certain cases with SCFE [137].

Increase width of the symphysis pubis and unaffected hip joint cartilage space has been observed [138], suggesting an abnormality of cartilage formation or maturation on a macroscopic level rather than a microscopic level. Skewness of the symphyseal joint has also been described on standing radiographs of the pelvis [139].

**Other Nonskeletal Associations**

Immune complexes in the synovial fluid and synovium of the hip, but not in the serum, have been found in a high percentage of SCFE cases [140, 141]. This is different from the synovitis of the knee or hip caused by other disorders where immune complexes are rarely found. Fluorides in the water, used to reduce dental caries, also strengthen the physes and metaphyseal bone; however, no difference in the incidence of SCFE was seen in children with or without fluoride exposure [142].

## 4. Conclusion

The current incidence of SCFE ranges from 0.33/100,000 to 24.58/100,000 children 8 to 15 years of age, depending upon gender and ethnicity. There is significant variability within racial groups and the relative frequency, relative to Caucasians at 1.0, is 5.6 for Polynesians, 3.9 for Blacks, and 2.5 for Hispanics. The average age is 12.0 years for boys and 11.2 years for girls; obese children present earlier than light weighted children. The physiologic age range during which SCFE occurs is less variable than the chronologic age range. The average symptom duration for stable SCFEs is 4 to 5 months. Although there is a general correlation between symptom duration and SCFE severity, there is considerable variability. The majority of children are obese with >50% of children >95th percentile weight for age; the average BMI in SCFE children is 25–30 kg/m^2^ or >85th percentile. The onset of SCFE is in the summer months in latitudes north of 40°N. Bilaterality ranges from 18 to 50% and is more frequent in Africans compared to Hispanic, White, and Indo-Malay peoples. In children with bilateral involvement, 50–60% present with simultaneous SCFEs; 80–90% of those who present with a unilateral SCFE and subsequently develop a contralateral SCFE do so within 18 months after the first SCFE. The age at presentation is younger for those who present with a unilateral SCFE and develop contralateral involvement compared to those who do not develop a contralateral SCFE. The age-weight, age-height, and height test are useful to differentiate between an idiopathic and atypical SCFE.

## Figures and Tables

**Table 1:** Incidence of slipped capital femoral epiphysis*.

Study	Year	City, Country	Region	Ethnicity	Incidence
Kelsey et al. [37]^⋀^	1970	Connecticut and New Mexico		All: <25 years old	
				Connecticut	3.41
				New Mexico	0.71
		Connecticut and New Mexico		All age restricted	
				Connecticut	10.08
				New Mexico	2.13
		Connecticut		White	
				Male	4.74
			North America	Female	1.64
				African	
				Male	7.79
				Female	6.68
				White	
				Urban	3.19
				Other	3.19
				African	
				Urban	7.95
				Other	1.35

Benson et al. [32]	2008	New Mexico	North America	All	6.05
				White	2.59
				Hispanic	5.49
				Native American	5.49
				African	11.57

Lehmann et al. [31]	2006	United States	North America	All	10.80
				Male	13.35
				Female	8.07
				White	6.24
				Hispanic	15.80
				Native American	5.13
				African	24.58
				Asian/Pacific Islanders	10.11
				Northeast	17.15
				Midwest	7.69
				South	8.12
				West	12.70

Larson et al. [39]	2010	Minnesota	North America	All	8.8

Jerre et al. [42]	1996	Gothenburg, Sweden	Scandinavia	All	7.1
				Male	9.0
				Female	5.1

Henrikson [43]	1969	Gothenburg, Sweden	Scandinavia	All	8.2

Hägglund et al. [34]	1984	Southern Sweden	Scandinavia	All	
				Male	7.1
				Female	5.3
				Rural	
				Male	7.5
				Female	2.2
				Urban	
				Male	4.8
				Female	3.8

Murray and Wilson [40]	2008	Scotland	Europe	All	
				1981	3.78
				2000	9.66

Noguchi and Sakamaki [38]	2002	Japan	Asia	All	1.51
				Male	2.22
				Female	0.76

Lim et al. [33]	2008	Singapore	Asia	All	1.2

Ninomiya et al. [36]	1976	Japan	Asia	All	0.25

Song et al. [44]	2009	Korea	Asia	All	0.33
				Male	0.50
				Female	0.14

^⋀^the overall breakdowns by gender and race (unrestricted) are given as incidence per 100,000 for all <25 years of age, while the other numbers are for boys 10–17 years old and girls 8–15 years old.

*incidence for children 8–15 years of age.

**Table:** (a)

Study	Year	City, Country	Region	Race	RRF
Kelsey et al. [37]	1970		North America	African	2.31
				White	1.0

Bosch et al. [32]	2008	New Mexico	North America	African	4.47
				Hispanic	2.12
				Native American	2.12
				White	1.0

Lehmann et al. [31]	2006	United States	North America	African	3.94
				Hispanic	2.53
				Asian/Pacific Islanders	1.62
				White	1.0

Stott and Bidwell [29]	2003	New Zealand	Australia/NZ	New Zealand Maori	5.6
				Pacific	4.2
				White	1.0

Loder [30]	1996	Worldwide		NA/Pacific Islanders	4.5
				African	2.2
				Hispanic/Native Am	1.05
				White	1.0
				Indo-Malay	0.5
				Indo-Med	0.1

^⋀^using Caucasian as a baseline frequency of 1.0.

**Table:** (b)

Study	Year	City, Country	Region	Race	RRF

Lim et al. [33]	2008	Singapore	Asia	Indian	9.6
				Mixed	3.0
				Malay	2.8
				Chinese	1.0

^+^using Chinese as a baseline frequency of 1.0.

**Table 3:** Age and gender in 4343 children with SCFE.

Study	Year	City/State/Country	Region	Race	Age	Age-M	Age-F	M	%M	F	%F
Stott and Bidwell [29]	2003	Auckland, New Zealand	Australia/NZ	All	11.82	12.46	11.05	115	54.5	96	45.5
				White	12.33	13.00	11.59	30	52.6	27	47.4
				Maori	11.51	12.18	10.69	33	55.0	27	45.0
				Pacific Islanders	11.65	12.24	10.92	49	55.1	40	44.9

Noguchi and Sakamaki [38]	2002	Japan	Asia	Indo-Malay (Japanese)	11.73	11.83	11.42	237	75.5	77	24.5
Jerre et al. [42]	1996	Gothenburg, Sweden	Scandinavia	White	13.07	13.60	12.10	113	64.6	62	35.4
Henrikson [43]	1969	Gothenburg, Sweden	Scandinavia	White	12.91	13.50	11.80	53	65.4	28	34.6
Hägglund et al. [34]	1984	Southern Sweden	Scandinavia	White	12.86	12.20	14.40	372	69.9	160	30.1

Loder [30]	1996	Worldwide	Worldwide	White	12.95	13.60	12.10	435	56.7	332	43.3
				African	12.88	13.80	11.70	225	56.1	176	43.9
				Amerindian	12.80	13.40	11.90	164	60.3	108	39.7
				Indo-Malay	12.09	12.30	11.50	89	74.2	31	25.8
				NA/PI	11.85	12.00	11.70	17	50.0	17	50.0
				Indo-Med	12.99	13.20	11.00	19	90.5	2	9.5
				All	12.88	13.50	12.00	959	58.8	671	41.2

Aronson and Loder [45]	1992	Detroit, Michigan	North America	African	12.53	13.42	11.08	46	62.2	28	37.8
Ninomiya et al. [36]	1976	Eastern Japan	Asia	Indo-Malay (Japanese)	12.49	12.75	11.00	58	85.3	10	14.7
Loder et al. [46]	2006	Michigan and Indiana	North America	All	12.60			159	65.4	84	34.6
Lim et al. [33]	2008	Singapore	Asia	All	12.20	12.40	11.10	53	80.3	13	19.7
Bosch et al. [32]	2008	New Mexico	North America	All	12.20	12.50	11.50				
Burrows [47]	1957	London, England	Europe		14.70	16.53	11.95	60	60.0	40	40.0
Carliozet al. [48]	1984	Paris, France	Europe		13.19	14.00	12.50	31	46.3	36	53.7
Aronson and Carlson [13]	1992	Detroit, Michigan	North America	All				31	70.5	13	29.5
Aronson et al. [49]	1992	Detroit, Michigan	North America	All	12.85	13.50	11.70	35	63.6	20	36.4
Carney et al. [26]	1991	Iowa	North America		12.98	13.40	11.30	99	79.8	25	20.2
Dreghorn et al. [50]	1987	Glasgow, Scotland	Europe		12.98	13.50	12.25	45	58.4	32	41.6
Kulick and Denton [51]	1982	New York	North America	All	12.62	13.00	12.00	58	61.7	36	38.3
Weiner et al. [35]	1984	Akron, Ohio	North America	All	13.05	13.40	12.20	113	71.1	46	28.9
Koval et al. [52]	1989	New York	North America	All	12.10			37	61.7	23	38.3
Song et al. [44]	2009		South Korea	Indo-Malay	12.67	12.83	12.00	175	75.8	56	24.2

Averages					**11.96**	**12.05**	**11.21**	**2806**	**64.3**	**1556**	**35.7**

The averages are weighted to account for the number of children in each series. The overall age was used in those series where ages are given by ethnicity [29, 30].

**Table 4:** Symptom duration in 2482 children with SCFE.

Study	Year	City/State/Country	Region	Number of Patients	Race	Sx Dur Males	Sx Dur Females	Sx Dur Combined
Hägglund et al. [34]	1984	Southern Sweden	Scandinavia	532	White	4.8	3.4	4.4

Loder [30]	1996	Worldwide	Worldwide	767	White	4.1	3.8	4.0
				401	African	4.9	3.6	4.3
				272	Amerindian	4.3	3.8	4.1
				120	Indo-Malay	5.1	5.5	5.2
				34	NA/PI	2.4	2.5	2.5
				21	Indo-Med	3.7	6.25	3.9
				1630	All	4.4	3.7	4.1

Loder et al. [46]	2006	Michigan and Indiana	North America	243	All			5.2
[50]	1987	Glasgow, Scotland	Europe	77	All			3.8

Totals				**2482**		**4.5**	**3.6**	**4.3**

The symptom duration averages are weighted to account for the number of children in each series. The overall symptom duration was used in the series where symptom duration is given by ethnicity [30].

**Table 5:** Laterality in 3037 children with SCFE.

Study	Year	City/State/Country	Region	Race	U	%U	B	%B	R	%R	L	%L
Stott and Bidwell [29]	2003	Auckland, New Zealand	Australia/NZ	All	64	55.7	51	44.3	22	34.4	42	65.6
				White	28	49.1	29	50.9	10	35.7	18	64.3
				Maori	36	60.0	24	40.0	8	22.2	28	77.8
				Pacific Islanders	47	52.8	42	47.2	18	38.3	29	61.7

Noguchi and Sakamaki [38]	2002	Japan	Asia	Indo-Malay (Japanese)	270	86.0	44	14.0	127	47.0	143	53.0
Jerre et al. [42]	1996	Gothenburg, Sweden	Scandinavia	White	173	98.9	2	1.1	69	39.9	104	60.1
Hägglund et al. [34]	1984	Southern Sweden	Scandinavia	White	451	84.8	81	15.2	171	33.5	339	66.5

Loder [30]	1996	Worldwide	Worldwide	White	663	83.2	134	16.8	239	37.8	394	62.2
				African	264	65.8	137	34.2	109	41.3	155	58.7
				Amerindian	227	83.5	45	16.5	96	42.5	130	57.5
				Indo-Malay	98	81.7	22	18.3	49	50.0	49	50.0
				NA/PI	21	61.8	13	38.2	8	38.1	13	61.9
				Indo-Med	13	61.9	8	38.1	5	38.5	8	61.5
				All	1267	77.7	363	22.3	510	40.3	756	59.7

Loder et al. [46]	2006	Michigan and India	North America	All	149	61.3	94	38.7	63	42.3	86	57.7
Lim et al. [33]	2008	Singapore	Asia	All	42	79.2	11	20.8	25	45.5	30	54.5
Burrows [47]	1957	London, England	Europe		77	77.0	23	23.0	29	37.7	48	62.3
Carlioz et al. [48]	1984	Paris, France	Europe		54	80.6	13	19.4	22	40.7	32	59.3
Aronson and Carlson [13]	1992	Detroit, Michigan	North America	All	30	68.2	14	31.8				
Aronson et al. [49]	1992	Detroit, Michigan	North America	All	30	54.5	25	45.5				
Carney et al. [26]	1991	Iowa	North America		93	75.0	31	25.0				
Dreghorn et al.[50]	1987	Glasgow, Scotland	Europe		58	75.3	19	24.7				
Kulick and Denton [51]	1982	New York	North America	All	63	67.0	31	33.0				
Koval et al. [52]	1989	New York	North America	All	29	59.2	20	40.8	36	45.0	44	55.0
Song et al. [44]	2009		South Korea	Indo-Malay	187	81.0	44	19.0	77	41.2	110	58.8

Totals					**3037**	**77.8**	**866**	**22.2**	**1151**	**39.9**	**1734**	**60.1**

**Table 6:** The age-weight or age-height test in children with SCFE is determined by graphically visualizing 6 groupings (or “cells”) of children with SCFE: cell 1, <10 years of age and ≥50th percentile in weight/height; cell 2, ≥10, ≤16 years of age and ≥50th percentile in weight/height; cell 3, >16 years of age and ≥50th percentile in weight/height; cell 4, <10 years of age and <50th percentile in weight/height; cell 5, ≥10, ≤16 years of age and <50th percentile in weight/height; and cell 6, >16 years of age and <50th percentile weight/height. Children in cells 3–6 (bold print) are considered to have a positive age-weight or age-height test; children in cells 1 and 2 (italic calligraphy) are considered to have a negative age-weight or age-height test.

Weight or height percentile	Age of patient with SCFE		
	<10 years	10–16 years	>16 years
≥50th	*Cell 1 (− test)*	*Cell 2 (− test)*	**CELL 3 (+ TEST)**
<50th	**CELL 4 (+ TEST)**	**CELL 5 (+ TEST)**	**CELL 6 (+ TEST)**

**Table 7:** Familial and genetic studies of SCFE.

Study	Year	Type of series	Number of cases	% familial incidence	Postulated inheritance	HLA Phenotype
Thrap-Meyer [64]	1940	Case report	Father and son	—	—	—
Irwin [65]	1946	Case report	Father and 2 twin sons	—	—	—
Smith [66]	1955	Case report	2 brothers	—	—	—
Wilson et al. [67]	1965	Case series	12 of 240 cases	5%	—	—
Rennie [68]	1967	Case reports	12 children, 8 different families	7%	Recessive with low penetrance	—
Ochsner et al. [69]	1977	Case report	10 members of one family	—	Autosomal dominant with variable penetrance	—
Gorin [70]	1977	Case report	Identical twins	—	—	
Rennie [71]	1982	Retrospective review	214	14.5%	Autosomal dominant with variable penetrance	—
				18.8% for osteoarthritis		
Hägglund et al. [72]	1986	Consecutive case series	50 (40 families)	8.1% in 1st degree relatives	—	—
Hägglund and Hansson [73]	1986	Case report, 3 generations	3 cases, 1 family	—	Autosomal dominant with variable penetrance	—
Gajraj [74]	1986	Case report, identical twins	1 family, identical twins	—	—	A11, B12
Mullaji et al. [75]	1993	HLA testing of SCFE patients	30 patients	—	—	B27 in 20%, > than in controls
Montskó and de Jonge [76]	1995	Case report	1 family father and 5 siblings (3 M, 2 F)	—	—	—
Moreira et al. [77]	1998	Case report	1 family, 4 cases	—	Autosomal dominant	—
Diwan et al. [78]	1998	Case report	1 family, 2 generations	—	—	—
Bednarz and Stanitski [79]	1998	Case report	Identical twins	—	—	Twin 1: A2, 26, B51, 60, Bw4/6
						Twin 2:A2,24, B51/60, Bw4/6
Allen and Calvert [80]	1990	Case report	Identical twins	—	—	A2, B12
Günal and Ateş [81]	1997	Case series	6 patients	—	—	DR4 common to all; no other common phenotypes
Wong-Chung et al. [82]	2000	Random case series	7 cases, 6 M, 1 F	2 were brothers	—	No common phenotypes
Siemon et al. [83]	2001	Case report	Identical twins	2 brothers	—	A2
Loder et al. [84]	2005	Case series	9 cases—Amish	39%		—
Sebastianowicz et al. [85]	2005	Case report	Identical twins	2 brothers	—	—
Flores et al. [86]	2006	Case report	Identical twins	—	—	A2, 23 (9), B44 (12), 38 (16) Bw4 Cw4, 12 DR103, DQ5 (1)
Lim et al. [87]	2007	Case report	Two brothers	—	Multifactorial	—
